# Influence of health education based on the transtheoretical model on kinesiophobia levels and rehabilitation outcomes in elderly patients undergoing total knee arthroplasty

**DOI:** 10.1016/j.heliyon.2024.e32445

**Published:** 2024-06-07

**Authors:** Ling-Xia Song, Li Yang, Ying Li, Fu-Qi Lei, Yi Qin, Lian-Hong Wang, Yong-Mei Zhang

**Affiliations:** a Department of Orthopedics, Affiliated Hospital of Zunyi Medical University, Zunyi, 563003, China; b Department of Nursing, Affiliated Hospital of Zunyi Medical University, Zunyi, 563003, China

**Keywords:** Health education, Kinesiophobia, Knee function, Rehabilitation self-efficacy, Total knee arthroplasty, Transtheoretical model

## Abstract

**Objective:**

In this study, we evaluated the effectiveness of health education based on the transtheoretical model in reducing symptoms of kinesiophobia and enhancing rehabilitation outcomes among elderly patients post-total knee arthroplasty.

**Methods:**

Elderly patients post-knee replacement surgery were randomly divided into a control group, which received standard health education, and an experimental group, which received transtheoretical model-based health education. The intervention commenced on the day after surgery and continued for a duration of six months. Assessments of kinesiophobia scores, rehabilitation self-efficacy, and knee function were conducted before the intervention, and then at one, three, and six months postoperatively.

**Results:**

Between January 2022 and December 2022, 130 elderly patients who met the eligibility criteria were enrolled and subsequently randomly assigned into two groups of equal size. Comparable baseline characteristics were observed between the two groups The experimental group demonstrated lower kinesiophobia scores and higher scores in rehabilitation self-efficacy and knee function at one, three, and six months following surgery, compared to the control group.

**Conclusion:**

Health education based on a transtheoretical model reduces the symptoms of kinesiophobia and enhances rehabilitation self-efficacy and knee functions in elderly patients after knee replacement surgery.

## 1 Introduction

The annual incidence of knee osteoarthritis (OA) is on the rise, coinciding with global aging trends. As the foremost chronic degenerative joint disorder, knee OA is characterized by symptoms such as joint pain, swelling, and restricted mobility [1]. Based on the 2021 American Academy of Orthopedic Surgeons (AAOS) clinical practice guidelines [2], the prevalence of knee OA in patients over 60 years old worldwide exceeds 10 %, making it one of the most common diseases affecting the quality of life of the elderly.

Knee OA not only impairs knee function but also imposes an economic burden on patients' families and society [3]. Total knee arthroplasty (TKA) is the primary surgical intervention for managing end-stage knee osteoarthritis, providing pain relief, correction of joint deformities, and an enhancement in the quality of life [4]. Kinesophobia, defined as the fear of movement, frequently manifests as a complication subsequent to TKA. This fear of movement primarily stems from various factors such as postoperative pain, diminished balance capabilities, and heightened psychological distress experienced by patients.

Notably, pain emerges as the principal factor fostering the aversion of movement among patients. When faced with pain, patients typically resort to one of two coping strategies: acceptance of the pain or an overwhelming fear of it [5]. Pain may induce negative psychological states such as fear, anxiety, and depression among patients. Concurrently, apprehension toward pain and exercise precipitates incorrect behavioral responses in rehabilitation exercises, characterized by the absence of proper guidance and correction. Prolonged adherence to such maladaptive behavior may result in the development of apraxia syndrome, typified by compromised cognitive and physical functioning [6].

Reports indicate a prevalence of postoperative kinesophobia ranging from 37.1 % to 45.68 % among patients with TKA [7,8]. The presence of kinesiophobia during hospitalization significantly delays the initial mobilization of patients from bed and extends the overall duration of their hospital stay. Consequently, this issue increases medical costs and imposes a considerable economic burden on patients [9,10].

The Transtheoretical Model (TTM), introduced by psychologist Prochaska in the early 1980s, initially found extensive application in the fields of addictive behavior, mental health, and various other domains [11,12]. Currently, it is one of the foremost and systematic models for the advancement of health promotion theory. Rooted in social psychology theory and informed by behavior change theory, TTM involves a thorough examination of the current behavioral status of patients. Integrating principles from psychology, behavioral science, and nursing science, TTM develops personalized intervention strategies aimed at addressing patients' maladaptive behavior. It seeks to gradually aid patients in altering detrimental behavior and psychological states, establishing appropriate behavior patterns, enhancing their self-management capabilities, and achieving a positive prognosis [13,14]. The theory has five distinct stages: the pre-intention stage, the intention stage, the preparation stage, the action stage, and the maintenance stage [15].

TTM has been successfully applied to elderly patients with cognitive impairment [16], breast cancer [17], coronary heart disease [18], among others, yielding positive outcomes. In China, there are currently few reports on TTM-based health education for elderly patients who have undergone TKA. Despite previous research involving collaboration with rehabilitation therapists to devise management programs for patients with knee OA post-TKA, there remains a notable gap in nurse-led research focused on developing a TTM-based health education program that encompasses the characteristics of phobia and rehabilitation self-efficacy among patients. Moreover, there is a need to enhance the assessment indicators utilized. Kinesiophobia has been indicated to significantly impact rehabilitation and prognosis in patients who have undergone TKA [19], independent of other psychological variables [20]. The presence of kinesiophobia throughout the postoperative course of TKA has a significant impact on patient recovery.

In this study, health education based on TTM and social psychology theory was administered to patients who had undergone TKA. A phased intervention was tailored to the specific characteristics of kinesiophobia and the postoperative rehabilitation needs of the patients. The objectives of this approach were to reduce kinesiophobia, enhance rehabilitation self-efficacy, and facilitate the recovery of knee function.

## 2 Methods

The aim of this randomized controlled trial was to assess the impact of health education based on TTM on kinesiophobia levels and rehabilitation outcomes in patients following knee replacement surgery. The participants were elderly patients hospitalized in Guizhou Province who had undergone TKA within the orthopedic department of a tertiary hospital.

### 2.1 Inclusion and exclusion criteria

Inclusion criteria: participants were individuals diagnosed with osteoarthritis who had undergone TKA for the first time and were at least 60 years of age. These patients possessed mental clarity and the ability to communicate effectively both verbally and in writing. Eligible patients received detailed information about the trial's nature, objectives, potential benefits, risks, and alternative treatment options, ensuring they had a comprehensive understanding and could make informed decisions regarding their participation.

Exclusion criteria: patients with mental abnormalities or cognitive impairments who cannot communicate normally with doctors or nurses, or patients with severe diseases of vital organs (heart, liver, lungs, kidneys, etc.).

Finally, 130 patients who underwent TKA were enrolled in the study, encompassing 110 patients with isolated knee degeneration, 13 patients with sole rheumatoid arthritis, and 7 patients with a combination of knee degeneration and rheumatoid arthritis. Among these, 42 patients presented with hypertension, 21 patients with diabetes, 13 patients with cerebral infarction, 20 patients with osteoporosis, and 2 patients with coronary atherosclerosis as comorbidities. Ethical approval for the study was obtained from the local ethics committee, and written informed consent was obtained from all participants.

## 3 Grouping of study participants

Utilizing the random number generator feature in SPSS version 27.0, participants included in the study were allocated into two groups: a control group and an experimental group. The procedure involved assigning data numbers from 1 to 130, organizing the participants based on their order of admission, initiating the random number generator, adjusting the maximum and minimum values, and ultimately generating random numbers. The data numbers 1 and 2 were designated for the control and experimental groups, respectively, resulting in 65 cases per group. To preserve the integrity of the trial results, this study was conducted as a single-blind experiment.

## 4 Intervention implementation

### 4.1 Control group

(1) The nurses provided health education, medication guidance, and dietary care immediately after the patient was admitted, and relevant disease knowledge was provided.
(2) Throughout the hospitalization period, the specialized nurse explained the essential perioperative nursing principles and health advisories, encompassing pain management strategies, medication directives, rehabilitation protocols, and measures for preempting postoperative complications, all imparted on the subsequent day following the surgical procedure.
(3) Patients were advised to scan the QR code available in the department for perioperative knee care, enabling them to learn various rehabilitation training methods and precautions through video-based instruction. Guidance on rehabilitation training covered a broad spectrum of activities aimed at enhancing muscle strength, enhancing joint range of motion, increasing proficiency in transfers, augmenting weight-bearing capacity, and performing exercises that support daily living functionality. The rehabilitation protocol adopted a systematic methodology, starting with lower limb muscle strength exercises (encompassing ankle pumps and quadriceps exercises) for patients as soon as they recuperated from anesthesia. These exercises were to be executed in three daily sessions, each consisting of 50–100 repetitions. Starting on the first day after surgery, patients were instructed to undertake joint range of motion exercises, including knee bending and straight leg raises, organized into three daily sessions of 10 repetitions each. Following these initial exercises, patients advanced to training focused on enhancing their transfer abilities and weight-bearing capacities, tailored to their specific rehabilitation status. Throughout the rehabilitation process, a gradual approach was emphasized, with adjustments made to exercise intensity in response to patient condition and muscle fatigue levels. Physical therapists diligently monitored patient progress and delivered personalized exercise directives in accordance with observed progress. The physiotherapist closely oversaw the progress of the patient throughout hospitalization, administering tailored exercise instructions in response to individualized needs.
(4) At the time of discharge, the “Artificial Total Knee Arthroplasty Rehabilitation Training Guidebook” was issued to the patients, along with discharge information, such as disease-related knowledge, pain management, wound management, rehabilitation training, diet, daily life guidance, and precautions. Following discharge, the patients were instructed to visit their neighborhood community hospital for additional rehabilitation sessions, while a specialized nurse conducted telephone follow-up for a duration of one week. Suture removal took place at the outpatient department of the hospital 14 days post-surgery. Subsequent clinic appointments were scheduled at intervals of 1, 3, and 6 months post-operatively for comprehensive follow-up assessments, which were conducted by orthopedic surgeons and physiotherapists.

### 4.2 Experimental group

(1) We established a five-member health education team based on the TTM theory. The head nurse of the department was the team leader and responsible for overall management. The health education team comprised of one researcher, two orthopedic specialist nurses, one orthopedic doctor, and one physical therapist. The specialist nurses and research team jointly oversaw the execution of the health education program, conducted follow-up surveys, and maintained comprehensive records.
(2) Pre-intention and intention phases: To foster intrinsic motivation in the rehabilitation endeavors of patients and cultivate adherence to rehabilitation protocols with confidence, specialist nurses engaged in interventions prior to and on the day of the operation, with each intervention spanning a duration of 20 min. These nurses conducted assessments of both the patients and their families' understanding of the disease and pain management, aiming to build a foundation of mutual trust. Subsequently, they assessed the levels of kinesiophobia and the readiness of the patients to partake in rehabilitation training. Patients were encouraged to assess and articulate the underlying causes of their kinesiophobia. Based on these assessments, nurses informed patients of the potential risks associated with kinesiophobia, tailored to their specific level of fear. Additionally, they supported patients in uncovering their internal motivation for initiating and sustaining behavior change. In addition, they encouraged patients to overcome the psychological barriers of kinesiophobia, assisted them in developing a correct perception of pain, to recognize the significance of early rehabilitation training, take the initiative to overcome kinesiophobia, and actively engage in rehabilitation training. The primary intervention strategies included the distribution of a manual on the management of postoperative kinesiophobia and one-on-one explanations to ensure that patients mastered pain self-management and nursing knowledge. Following the intervention, the specialist nurse assessed the patients to ascertain their comprehension of concepts pertinent to fear of movement and rehabilitation training. In cases where patients demonstrated incomplete mastery of the subject matter, the intervention persisted until patients attained a thorough and accurate understanding of both the fear of movement phenomenon and rehabilitation training principles.
(3) Preparation phase: The TTM Health Education team crafted personalized, phase-wise health education programs with the aim to bolster the motivation and self-assurance of patients in adhering to prescribed medical rehabilitation regimens., Orthopedic therapists, specialist nurses, and physical therapists collaboratively undertook comprehensive assessments of the post-operative status of patients within a 6-h timeframe. They assessed the potential challenges patients might face in altering their kinesiophobia-related behavior. Together, they formulated tailored health education programs with the patients, aiming to equip them with effective functional exercise techniques and strategies for managing pain during physical activity. At this juncture, through methods such as conversation, explanation, discussion, and introspection, the health education team aided patients in recognizing and reformulating a correct psychological understanding of pain that might arise during daily activities and functional exercises. Through repeated guidance, any misconceptions regarding pain were addressed and rectified, with supplementary instructions being offered as necessary. During the preparatory phase, the assessment and intervention process extended for a duration of 20 min. Subsequent to the intervention, patients underwent assessment to ascertain the establishment of confidence regarding pain management and rehabilitation behaviors. In cases where patients did not achieve the desired confidence level, the intervention persisted until the specified level was attained, ensuring that patients were adequately equipped to navigate pain management and rehabilitation endeavors with assurance.
(4) Action phase: The rehabilitation behavior change program was executed with a daily emphasis on encouraging patients to achieve predetermined goals. Collaboratively, specialist nurses and physical therapists conducted assessments of the pain levels, risk of movement phobia, and functional exercise capacity of the patients from the initial day to the fifth day post-surgery. Tailoring the rehabilitation regimen to individual patient factors such as motor phobia severity, symptom awareness, recovery trajectory, and cooperation, patients were guided to engage in rehabilitation activities thrice daily, with each session extending approximately 30 min. These sessions focused on enhancing muscle strength, enhancing joint mobility, increasing transfer proficiency, training weight-bearing capacity, and performing exercises that support daily living functionality following surgery. During the rehabilitation process, patients were provided with targeted guidance aimed at maintaining joint function, reducing fears associated with pain, and ensuring the successful completion of prescribed rehabilitation exercises. Patients were encouraged to record the location, cause, intensity, duration, and impact of daily pain in a pain diary [21]. Furthermore, patients received assistance in surmounting psychological obstacles and managing negative emotions through the utilization of calming music, muscle relaxation training, and meditation techniques, enabling them to psychologically reconcile with the outcomes of surgery. A quantitative record table for rehabilitation training was established, and patients were encouraged to meticulously log the content, duration, intensity, and impact of their daily rehabilitation exercises. This approach aimed not only to monitor progress but also to reinforce the patients' engagement and ownership of their rehabilitation journey. At this phase, the health education team persistently focused on correcting misconceptions related to kinesiophobia, providing repeated guidance to address any deficiencies. They supported patients in overcoming their fear of movement, assessed the patients' understanding and application of rehabilitation training content, and aided in fostering a habit of regular exercise among the patients. Following discharge, specialist nurses and physical therapists provided ongoing guidance and supervision to patients via online video platforms, facilitating the completion of daily rehabilitation plans.
(5) Maintenance phase: The health education team continued to strengthen supervision and management to consolidate the behavior change. Weekly telephone follow-ups and micro-video feedback sessions were conducted to maintain engagement and monitor progress. Sutures were removed during a visit to the outpatient clinic 14 days post-surgery, with subsequent monthly follow-ups for six months. Through these methods, the health education team dynamically assessed changes in patients' kinesiophobia levels and their compliance with the rehabilitation training regimen. This ongoing assessment facilitated the provision of personalized and targeted rehabilitation guidance, designed to meet the evolving needs and challenges of each patient throughout their recovery. Physical therapists and specialist nurses conducted weekly micro-video interventions for a duration of 20 min each, extending over a period of 6 months following discharge. During these interventions, they documented patient adherence to rehabilitation exercises and confirmed the outcomes resulting from the observed behavioral changes.

### 4.3 Research tools

Evaluations were carried out with a comprehensive array of instruments, including the General Information Questionnaire, the Tampa Scale of Kinesiophobia (TSK), the Self-Efficacy for Rehabilitation Outcome Scale (SER), and the Hospital for Special Surgery knee score (HSS).

(1) General Information Questionnaire: After a thorough examination of pertinent literature and engaging in group discussions, we developed a questionnaire composed of two sections: one focusing on population sociology and the other on disease-related data. The section dedicated to population sociology encompasses variables such as gender, age, body mass index (BMI) value, education level, occupation, pre-existing conditions (such as hypertension, diabetes, etc.), methods of medical expense payment, among other factors.
(2) Chinese version of Simplified Kinesiophobia Scale: The scale was originally compiled by Woby et al. [22] and was modified by Cai et al. [23] The Chinese version of the scale demonstrated a Cronbach's coefficient of 0.74, indicative of acceptable internal consistency, along with a retest reliability of 0.86, denoting strong stability over time. Comprising a total of 17 items, each item was assessed using a Likert 4 scale, spanning from 1 (strongly disagree) to 4 (strongly agree), with reversed scoring applied to items 4, 8, 12, and 16. Consequently, the total score ranged from 17 to 68 points. A diagnosis of kinesiophobia was established if the total score surpassed 37 points, as outlined in supplementary file 1.
(3) Self-Efficacy for Rehabilitation Outcome Scale (SER): This scale was developed by Waldrop et al. [24] and translated into Chinese by Wang et al. [25] It consists of 2 dimensions and a total of 12 items, namely rehabilitation exercise self-efficacy (items1–5) and coping self-efficacy (6–12). The Likert grading method is used in this scale. The total score is the sum of each item's score. It is categorized from 0 to 10 levels, with 0 indicating complete incapability and 10 indicating completely capable. The total score ranges from 0 to 120. The greater the score, the greater the level of self-efficacy. The scale exhibits a Cronbach's coefficient of 0.942, underscoring its commendable reliability and validity, as documented in supplementary file 2.
(4) The Hospital for Special Surgery knee score (HSS Scale), devised by Insall et al. at the American Hospital for Special Surgery, consists of seven questions [26]. Among these, scores for six questions are used to gauge various dimensions such as pain (30 points), joint function (22 points), range of motion (18 points), muscle strength (10 points), flexion deformity (10 points), and joint stability (10 points). Conversely, the score from the remaining question, which pertains to the use of a walker, varus deformity, valgus deformity, or extension limitation, is deducted from the overall score. The total HSS score ranges from 0 to 100 points. Poor performance is indicated by a score of 59 points or less, moderate performance by 60–69 points, good performance by 70–84 points, and excellent performance by 85 points or more. Higher scores correlate with enhanced knee joint functionality and overall better outcomes, as delineated in supplementary file 3.

### 4.4 Data analysis

The determination of the study's sample size followed a methodical approach, employing the estimation technique tailored for the comparison of two independent sample means. With the selection of a significance level (α) set at 0.05, power (β) at 0.10, and the adoption of a two-sided test, critical values were subsequently derived in accordance with the specified parameters: t_0.05/2_ = 1.96, t_0.1_ = 1.28. After reviewing pertinent literature, a standard deviation (σ) of 0.76 and a mean difference (δ) of 0.48 were identified [27]. Subsequently, applying the formula, a requirement of 54 cases per group was established. Accounting for a projected dropout rate of 20 %, the final determined sample size amounted to 65 cases for each group, thus culminating in a total sample size of 130 cases.

SPSS27.0 was used to organize and analyze the data. The inspection threshold was established at 0.05. The measurement data are expressed as the mean and standard deviation (‾*x* ***± s***), and the independent-samples *t*-test was utilized to compare the two groups. The counting data are expressed in terms of frequency and percentage, and the chi-squared test was utilized to compare between the groups. ANOVA for repeated measurements was utilized for data with normal distribution and homogeneous variance. Given the normal distribution and homogeneity of variance observed in the study data, ANOVA was used for repeated measurements of statistical analysis. Before conducting the ANOVA test, the sphericity test was administered. In the event of rejecting the null hypothesis, the multivariate test was employed. In cases where ANOVA for repeated measurements was utilized, assessments of both between-group and intra-group comparisons were conducted in the presence of an interaction effect. For comparison purposes, one-way ANOVA was utilized. Subsequently, upon detecting significant differences, the LSD method was employed for post-hoc multiple comparisons.

## 5 Results

This study included a total of 130 elderly patients who had undergone knee replacement surgery, with 65 cases each in the control group and in the experimental group. During the study, 1 patient from the control group was lost to follow-up post-discharge, while 2 patients were transferred to an alternate department for treatment. Additionally, 2 patients in the experimental group demonstrated inadequate compliance. Consequently, data from a total of 125 patients were collected and documented for analysis. In terms of gender, age, BMI, education level, and underlying diseases, there were no significant differences between the two groups at baseline (*P* > 0.05), indicating comparability (Table 1).

**Table 1:** Comparison of general data between the two groups.

Item		Experimental group (n = 63)	Control group (n = 62)	Statistical values (χ^*2*^*/F*)	*P*
Gender	Male	24 (38.1)	28 (45.2)	0.642^a^	0.423
	Female	39 (61.9)	34 (54.8)		
Age		71.22 ± 5.091	73.73 ± 4.771	0.059^b^	0.445
BMI		23.30 ± 2.318	23.19 ± 2.289	0.035^b^	0.852
Education level	Junior high school and below	16 (25.4)	17 (27.4)	0.732^a^	0.693
	High school or technical secondary school	28 (44.4)	23 (37.1)		
	Junior college and above	19 (30.2)	22 (35.5)		
Occupation	Civil servant or worker	12 (19.1)	18 (29.1)	3.743^a^	0.154
	Farmer	25 (39.7)	28 (45.1)		
	Merchant or other	26 (41.2)	16 (25.8)		
Underlying diseases	Yes	12 (19.0)	16 (25.8)	0.821^a^	0.365
	No	51 (81.0)	46 (74.2)		
Medical expenses payment method	Employee health insurance	9 (14.3)	11 (17.7)	2.010^a^	0.570
	Resident health insurance	20 (31.7)	13 (21.0)		
	New rural cooperative medical system	22 (34.9)	26 (41.9)		
	Others	12 (19.1)	12 (19.4)		

Note1.

a Indicates χ^*2*^.

b Indicates *F*.

**Fig. 1:**
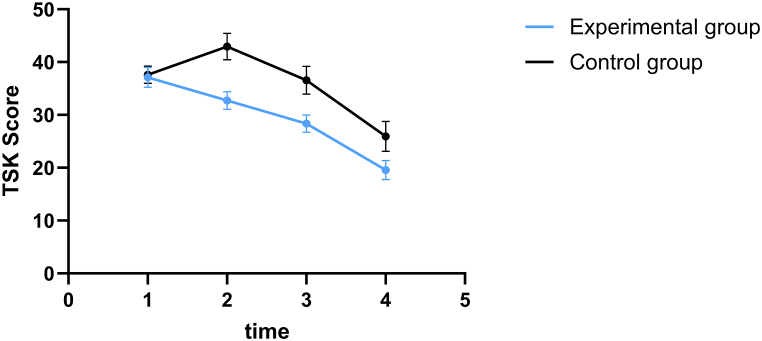
Variation trend of Tampa Scale of Kinesiophobia (TSK) scores in the two groups at four different time points.

There was no significant difference between the preoperative TSK scores of the two groups (*P* > 0.05). At the 1st, 3rd, and 6th month after surgery, the TSK scores of the experimental group were significantly lower than those of the control group (*P* < 0.05). The differences in TSK scores between the two groups at the three pre-intervention and post-intervention time points were statistically significant (*P* < 0.05) (Table 2, Fig. 1).

**Table 2:** Comparison of the kinesiophobia (TSK) scores (score, ‾***x* ± s**) of the two groups.

Group	Cases	Before surgery	1 month after surgery	3 months after surgery	6 months after surgery	F value of time effect	F value of treatment effect	F value of interaction effect
Experimental group	63	37.10 ± 1.88	32.71 ± 1.70^a^	28.19 ± 1.70^a^	19.83 ± 1.78^a^	1379.01^a^	791.25^a^	119.57^a^
Control group	62	37.60 ± 1.61	42.95 ± 2.50	36.37 ± 2.29	26.31 ± 2.89			

Note.

a Indicates that the difference between the experimental group and the control group was statistically significant (P < 0.05).

**Fig. 2:**
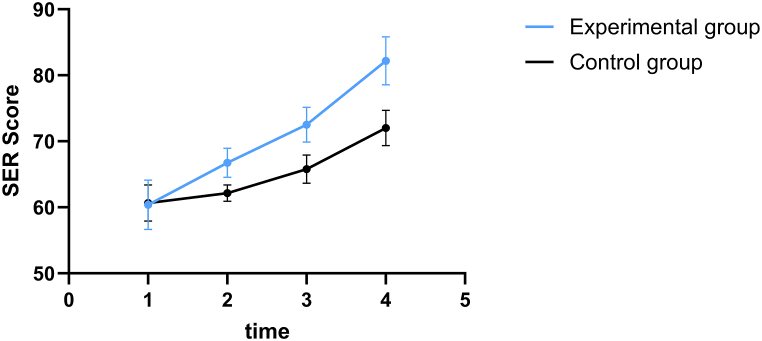
Variation trend of Self-Efficacy for Rehabilitation Outcome Scale (SER) scores in the two groups at four different time points.

The preoperative SER scores of both groups did not differ significantly (*P* > 0.05). At the 1st, 3rd, and 6th month after surgery, the SER scores of the experimental group were significantly higher than those of the control group (*P* < 0.05). At all three time points before and after the intervention, there was a statistically significant difference in SER scores between the two groups (*P* < 0.05) (Table 3, Fig. 2).

**Table 3:** Comparison of the rehabilitation self-efficacy (SER) scores (score, ‾**x ± s**) of the two groups.

Group	Cases	Before surgery	1 month after surgery	3 months after surgery	6 months after surgery	F value of time effect	F value of treatment effect	F value of interaction effect
Experimental group	63	60.40 ± 3.75	66.76 ± 2.22^a^	72.51 ± 2.63^a^	82.19 ± 3.64^a^	963.71^a^	340.00^a^	89.78^a^
Control group	62	60.65 ± 2.75	62.16 ± 1.23	65.79 ± 2.15	72.03 ± 2.69			

Note.

a Indicates that the difference between the experimental group and the control group was statistically significant (P < 0.05).

**Fig. 3:**
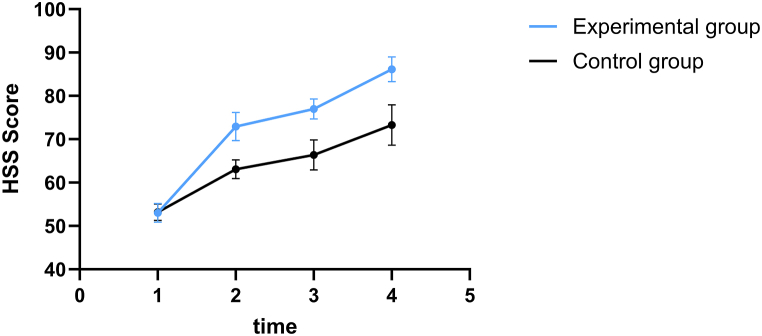
Variation trend of Hospital for Special Surgery knee score (HSS) scores in the two groups at four different time points.

There was no significant difference between the preoperative HSS scores of the two groups (*P* > 0.05). At the 1st, 3rd, and 6th month after surgery, the HSS scores of the experimental group were significantly higher than those of the control group (*P* < 0.05). At the three time points before and after the intervention, there was a statistically significant difference in HSS scores between the two groups (*P* < 0.05) (Table 4, Fig. 3).

**Table 4:** Comparison of the knee function (HSS) scores (score, ‾**x ± s**) of the two groups.

Group	Cases	Before surgery	1 month after surgery	3 months after surgery	6 months after surgery	F value of time effect	F value of treatment effect	F value of interaction effect
Experimental group	63	53.00 ± 2.20	72.92 ± 3.23^a^	76.98 ± 2.32^a^	86.14 ± 2.87^a^	4191.40^a^	610.53^a^	237.14^a^
Control group	62	53.19 ± 1.92	63.08 ± 2.17	66.37 ± 3.47	73.26 ± 4.68			

Note.

a Indicates that the difference between the experimental group and the control group was statistically significant (P < 0.05).

## 6 Discussion

Knee OA is a prevalent chronic musculoskeletal disease in the elderly, with TKA being the most effective treatment for end-stage osteoarthritis, rheumatoid arthritis, and other diseases [28]. With increasing incidence of knee OA, the overall demand for TKA is also increasing each year [29]. Rehabilitation following TKA is crucial to enhancing the prognosis of patients who have undergone TKA [30]. TKA is also of significant importance in alleviating knee OA symptoms and preventing disease progression. Nevertheless, a substantial proportion of patients who undergo TKA comprises elderly individuals characterized by limited understanding and self-management capabilities, often grappling with enduring pain. The surgical procedure itself is inherently traumatic, further compounded by patients' inadequate grasp of their ailment, leading to heightened fear of pain and apprehensions regarding further deterioration of knee function or surgical failure due to exercise. Consequently, patients are prone to develop kinesiophobia symptoms, resulting in low self-efficacy and rehabilitation compliance, which is not conducive to recovery [[31], [32], [33]]. It has been reported that patients with kinesiophobia firmly believe that exercise will exacerbate their body's pain and affect their postoperative rehabilitation [34].

Consequently, in this study, the TTM was applied, taking into account the kinesiophobia characteristics of the patients. Particular emphasis was placed on monitoring the evolving psychological dynamics of patients and deploying a staged approach to health education interventions, tailored to individual levels of kinesiophobia and cognitive capacity. This strategy aimed to prevent information overload among patients by disseminating educational content incrementally. Moreover, we iteratively refined and augmented the health education regimen, thereby bolstering patients' motivation to engage in rehabilitation exercises and adhere to prescribed treatment.

As per the findings of this study, TSK scores of the experimental group at 1st, 3rd, and 6th month after the intervention were significantly lower than those of the control group (*P* < 0.05). In addition, the TSK scores of the control group revealed an upward trend in the 1st month after surgery, followed by a downward trend in the 3rd and 6th month, indicating that the level of kinesiophobia in the control group was still high one month after surgery due to surgical trauma. At the 1st, 3rd, and 6th month after surgery, the TSK scores of the experimental group continued to decrease, indicating that TTM-based health education could reduce kinesiophobia in patients after TKA.

Based on the results, the rehabilitation self-efficacy and knee function scores in the experimental group were higher than those in the control group (*P* < 0.05), and the SER and HSS scores in the control group and experimental group indicated a downward trend in the 1st, 3rd, and 6th month following surgery. However, the experimental group revealed a greater decline than the control group, indicating that TTM-based health education can enhance the rehabilitation self-efficacy of patients and promote the recovery of knee function, consistent with previous studies [35,36]. The primary reason is that, during the process of implementing TTM-based health education, the health knowledge, rehabilitation self-efficacy, and confidence of patients in rehabilitation are consistently enhanced [37,38].

The purpose of the pre-intention phase and intention phase was primarily to clarify and answer patient questions, as well as to assist patients in developing a correct understanding of their disease, kinesiophobia, and rehabilitation training, so as to enhance their rehabilitation self-efficacy. In the preparation phase, a practical health education program was formulated with patients based on their characteristics, and the target values of health education are established. In the action phase, patients were encouraged to maintain a quantitative record sheet of rehabilitation training and a pain diary. This was relatively easy for patients to actively conduct and to self-evaluate their status, enabling them to intuitively understand the effect of their pain management and rehabilitation training. This not only helped patients make progress in stages and gain self-confidence and a sense of accomplishment in self-rehabilitation management, but it also enhanced their rehabilitation self-efficacy and rehabilitation outcomes. If there was a problem with the implementation of rehabilitation training, patients could consult at any time via phone and WeChat. For example, if a patient experienced knee pain or abnormal knee sounds during training, the patients promptly received guidance on the cause; they were encouraged to continue training, to reduce the pressure they experienced during the behavior change process, and family members were asked to participate in order to provide encouragement and social support.

In the maintenance phase, after discharge, regular telephone follow-up, outpatient follow-up, WeChat, video, and other online approaches were used to provide ongoing nursing care. Thus, the health education team was aware of the patients’ health behavior and the achievement of their goals and could consistently consolidate the transformation of the action stage using stimulation and control strategies, such as regular follow-up and video feedback. This enabled the patients to subtly achieve behavioral changes and cultivate habits, which ultimately promoted their recovery.

### 6.1 Limitations

The present study is subject to several limitations. Primarily, the sampled population may not adequately reflect the broader characteristics of elderly individuals with knee OA, potentially constraining the generalizability of health education initiatives based on TTM. Secondly, the outcomes concerning kinesiophobia symptoms and self-efficacy in this investigation could be susceptible to participant-reported subjective biases, potentially impacting the robustness of the findings. Despite employing uniform and standardized language to elucidate questionnaire items and uphold their precision, we acknowledge the necessity to strengthen the resilience of forthcoming investigations. Notably, augmenting the sample size is imperative to enhance statistical potency. Furthermore, endeavors should be directed toward mitigating the potential influence of educational background, personal values, and other socio-ecological variables that may sway patients' subjective judgments. Additionally, conducting higher-quality randomized controlled trials is warranted to advance the methodological rigor of future studies.

## 7 Conclusion

The health education program founded on TTM emerges as a straightforward, pragmatic, secure, and efficacious intervention in attenuating kinesiophobia following TKA. This approach not only augments self-efficacy in rehabilitation but also fosters the restoration of knee function among patients. Its versatility for implementation extends across hospital and community settings, providing a viable avenue to ameliorate symptoms and expedite postoperative recovery, particularly among elderly patients undergoing TKA.

## Ethics approval and consent to participate

This study was conducted with approval from the Ethics Committee (approval number: KLL-2020-325). This study was conducted in accordance with the declaration of Helsinki. Written informed consent was obtained from all participants.

## Consent for publication

Not applicable.

## Data availability statement

Data associated with this study has not been deposited into publicly available repository. Data will be made available on request.

## Funding

This work was supported by 10.13039/501100004001Science and Technology Fund Project of Guizhou Provincial Health Commission (No. gzwjkj2020-1-219) and also was supported by Zun City science and technology cooperation HZ word (2022) 217.

## CRediT authorship contribution statement

**Ling-Xia Song:** Writing – original draft, Funding acquisition, Formal analysis, Conceptualization. **Li Yang:** Writing – original draft, Methodology, Data curation, Conceptualization. **Ying Li:** Writing – review & editing, Methodology, Funding acquisition. **Fu-Qi Lei:** Methodology, Data curation. **Yi Qin:** Writing – review & editing, Formal analysis, Data curation. **Lian-Hong Wang:** Writing – original draft, Conceptualization. **Yong-Mei Zhang:** Writing – review & editing, Conceptualization.

## Declaration of competing interest

The authors declare that they have no known competing financial interests or personal relationships that could have appeared to influence the work reported in this paper.
