# Supplementary material for: Influence of health education based on the transtheoretical model on kinesiophobia levels and rehabilitation outcomes in elderly patients undergoing total knee arthroplasty
Source: Heliyon. 2024 Jun 7;10(12):e32445. doi: 10.1016/j.heliyon.2024.e32445 (PMC11225756; doi:10.1016/j.heliyon.2024.e32445)
Supplement: Multimedia component 1 [file mmc1.docx]

**Self-Efficacy for Rehabilitation Outcome Scale(TSK)**

|  | serious  opposition | opposition | agreement | complete agreement |
| --- | --- | --- | --- | --- |
| 1.If I exercise, I will be afraid to hurt myself. | 1 | 2 | 3 | 4 |
| 2.If I try to overcome it, the pain gets worse | 1 | 2 | 3 | 4 |
| 3.My body was telling me I was making a very dangerous mistake | 1 | 2 | 3 | 4 |
| 4.if I exercise,The pain will probably ease | 1 | 2 | 3 | 4 |
| 5.People didn 't pay enough attention to my health. | 1 | 2 | 3 | 4 |
| 6.The accident has put my body at risk in the future. | 1 | 2 | 3 | 4 |
| 7.Pain always means that the body has been hurt | 1 | 2 | 3 | 4 |
| 8.Just making the pain worse doesn 't mean they are dangerous. | 1 | 2 | 3 | 4 |
| 9.I'm afraid I might accidentally hurt myself | 1 | 2 | 3 | 4 |
| 10.Do not do more action, simple to keep careful is the safest thing I can do to prevent the deterioration of pain. | 1 | 2 | 3 | 4 |
| 11.I wouldn 't feel pain if there weren 't some potentially dangerous things going on inside me. | 1 | 2 | 3 | 4 |
| 12.I feel very painful, but if I am active, the situation will improve. | 1 | 2 | 3 | 4 |
| 13.The pain makes me know when to stop exercising to prevent injury | 1 | 2 | 3 | 4 |
| 14.It is really unsafe to be active like me. | 1 | 2 | 3 | 4 |
| 15.I ' m too vulnerable to do what ordinary people can do. | 1 | 2 | 3 | 4 |
| 16.Although some things have brought me a lot of pain. But I don 't think they 're dangerous. | 1 | 2 | 3 | 4 |
| 17.No one has to exercise in pain. | 1 | 2 | 3 | 4 |
