# Supplementary material for: Influence of health education based on the transtheoretical model on kinesiophobia levels and rehabilitation outcomes in elderly patients undergoing total knee arthroplasty
Source: Heliyon. 2024 Jun 7;10(12):e32445. doi: 10.1016/j.heliyon.2024.e32445 (PMC11225756; doi:10.1016/j.heliyon.2024.e32445)
Supplement: Multimedia component 2 [file mmc2.docx]

**Self-Efficacy for Rehabilitation Outcome** **Scale（SER）**

1. In the process of rehabilitation, I believe that I can do the exercise that needs to stretch my legs.

0 1 2 3 4 5 6 7 8 9 10

It cannot be done at all It can be done completely

2.In the process of rehabilitation, I believe that I can do the exercise that needs to lift my leg.

0 1 2 3 4 5 6 7 8 9 10

It cannot be done at all It can be done completely

3.In the process of rehabilitation, I believe that I can do the exercise that needs to bend my leg.

0 1 2 3 4 5 6 7 8 9 10

It cannot be done at all It can be done completely

4.In the process of rehabilitation, I believe that I can do the exercise that needs to stand up.

0 1 2 3 4 5 6 7 8 9 10

It cannot be done at all It can be done completely

5.In the process of rehabilitation, I believe that I can do the exercise that needs to walk.

0 1 2 3 4 5 6 7 8 9 10

It cannot be done at all It can be done completely

6.In the process of rehabilitation, I believe I can do all the required rehabilitation exercises.

0 1 2 3 4 5 6 7 8 9 10

It cannot be done at all It can be done completely

7.In the process of rehabilitation, I believe I can complete the daily plan of rehabilitation exercise.

0 1 2 3 4 5 6 7 8 9 10

It cannot be done at all It can be done completely

8.In the process of rehabilitation, even if I don 't understand how exercise is helpful to my rehabilitation, I can carry out rehabilitation exercise according to the requirements of medical staff.

0 1 2 3 4 5 6 7 8 9 10

It cannot be done at all It can be done completely

9.In the process of rehabilitation, I can still insist on doing rehabilitation exercise when I am depressed.

0 1 2 3 4 5 6 7 8 9 10

It cannot be done at all It can be done completely

10.In the process of rehabilitation, no matter how tired, I can still insist on doing rehabilitation exercise.

0 1 2 3 4 5 6 7 8 9 10

It cannot be done at all It can be done completely

11.In the process of rehabilitation, even if there have been other complications, I can still insist on doing rehabilitation exercise.

0 1 2 3 4 5 6 7 8 9 10

It cannot be done at all It can be done completely

12.In the process of rehabilitation, no matter how painful or uncomfortable the body is, I can still insist on doing rehabilitation exercises.

0 1 2 3 4 5 6 7 8 9 10

It cannot be done at all It can be done completely
