# Supplementary material for: Influence of health education based on the transtheoretical model on kinesiophobia levels and rehabilitation outcomes in elderly patients undergoing total knee arthroplasty
Source: Heliyon. 2024 Jun 7;10(12):e32445. doi: 10.1016/j.heliyon.2024.e32445 (PMC11225756; doi:10.1016/j.heliyon.2024.e32445)
Supplement: Multimedia component 3 [file mmc3.docx]

**Hospital for Special Surgery knee score(HSS Scale)**

**1.**Pain(30 points)

| No pain at any time | 30 |  |  |  |  |  |  |
| --- | --- | --- | --- | --- | --- | --- | --- |
| No pain when walking | 15 |  |  | No pain at rest | 15 |  |  |
| Mild pain while walking | 10 |  |  | Mild pain at rest | 10 |  |  |
| Moderate pain while walking | 5 |  |  | Moderate pain at rest | 5 |  |  |
| Severe pain while walking | 0 |  |  | Severe pain at rest | 0 |  |  |

2. Joint function(22 points)

| Unlimited walking and standing | 22 |  |  |  |  |  |  |
| --- | --- | --- | --- | --- | --- | --- | --- |
| Walking 2500 ~ 5000 meters and standing for more than half an hour | 10 |  |  | Walking in the house, no need for braces | 5 |  |  |
| Walking 500 ~2500 meters and standing for half an hour | 8 |  |  | Walking in the house requires braces | 2 |  |  |
| Walking less than 500 meters | 4 |  |  | Can go up the stairs | 5 |  |  |
| unable to walk | 0 |  |  | Can go upstairs, but need braces | 2 |  |  |

**3.**Range of motion(18 points)

| 8 degrees=1 point | The highest is 18 points. |  |  |
| --- | --- | --- | --- |

**4.**Muscle strength(10 points)

| excellent: resist resistance completely | 10 |  |  | average:drive joint activity | 4 |  |  |
| --- | --- | --- | --- | --- | --- | --- | --- |
| good:part against resistance | 8 |  |  | poor:can 't drive joint activity | 0 |  |  |

5.Flexion deformity(10 points) **6.**Joint stability(10 points)

| No congenital deformities | 10 |  |  | Normal | 10 |  |  |
| --- | --- | --- | --- | --- | --- | --- | --- |
| Less than 5 degrees | 8 |  |  | Mild instability 0 ~ 5 degrees | 8 |  |  |
| 5~10degrees | 5 |  |  | Moderate instability 5 ~ 15 degrees | 5 |  |  |
| More than 5 degrees | 0 |  |  | Severe instability more than 15 degrees | 0 |  |  |

7.Points reduction project

| Single stick | -1 |  |  | Straightening stagnate 5 degrees | -2 |  |  | Eversion every 5 degrees | -1X |  |  |
| --- | --- | --- | --- | --- | --- | --- | --- | --- | --- | --- | --- |
| Single crutch | -2 |  |  | Straightening stagnate10 degrees | -3 |  |  | Inverted every 5 degrees | -1X |  |  |
| Double crutches | -3 |  |  | Straightening stagnate 15 degrees | -5 |  |  |  |  |  | |
